# Promoting NK cell trafficking to improve therapeutic effect of NK cell therapy on osteosarcoma

**DOI:** 10.1186/2051-1426-3-S2-P24

**Published:** 2015-11-04

**Authors:** Yuanzheng Yang, Nancy Gordon, Eugenie S Kleinerman, Gangxiong Huang

**Affiliations:** 1 MD Anderson Cancer Center, Houston, TX, USA; 2 University of Texas MD Anderson Cancer Center, Houston, TX, USA

## Background

Despite the ability of NK cells to kill cancer cells, NK cell–based immunotherapy has resulted in limited clinical benefit. This is largely due to the poor capacity of adoptively transferred NK cells to home to tumors. We have shown that NK cells are able to kill osteosarcoma (OS) cells *in vitro* in a dose-dependent manner, indicating that recruiting more NK cells to OS lung metastasis *in vivo* may help to achieve greater therapeutic benefit. NK cell trafficking is mediated by specific chemokine receptors expressed on NK cells, but the specific receptors necessary for NK cell migration to solid tumors including OS are ill-defined.

## Methods

In this study, we demonstrate that the specific chemokine receptor, CXCR2, enhances the migration and infiltration of adoptively transferred NK cells to OS lung metastases in mice.

## Results

NK cells have low level expression of CXCR2 and less than 10% of NK cells isolated from human peripheral blood mononuclear cells are CXCR2^+^. However, selection for an enriched CXCR2^+^ NK cell population before NK cell transfer to mice bearing OS lung metastases demonstrated that CXCR2^+^ NK cells have increased tumor infiltration compared to control NK cells. Additionally, over-expression of CXCR2 on NK-92 (NK-92-CXCR2) cells promotes their migration to OS cell conditioned medium *in vitro*, and enhances their infiltration to lung metastases *in vivo*. Further, adoptive transfer of NK-92-CXCR2 resulted in improved therapeutic effect on established OS lung metastases.

## Conclusions

Given our demonstration of the critical role of CXCR2 in NK cell migration to tumors, our ongoing work aims to develop a strategy to promote NK cell trafficking to OS lung metastases by inducing the expression of CXCR2 on primary human NK cells.

